# Supplementary material for: Restrictive Cardiomyopathy is Caused by a Novel Homozygous Desmin (DES) Mutation p.Y122H Leading to a Severe Filament Assembly Defect
Source: Genes (Basel). 2019 Nov 11;10(11):918. doi: 10.3390/genes10110918 (PMC6896098; doi:10.3390/genes10110918)
Supplement: Supplementary file 1 [file genes-10-00918-s001.pdf]

Table S1. Overview about genetic non-synonymous, rare variants (AF<0.01) in the genome of III-1.

Gene	Transcript	Chromosome	Coordinate [1]	ID	Variant	Protein alteration	Type of variation	Allele frequency [2]	Zygosity	ACMG classification
TTN	NM_001267550.1	2	179414961	novel	C>C/G	p.G30535A	missense	0	heterozygous	VUS
TTN	NM_001267550.1	2	179568949	novel	C>C/T	p.V10050I	missense	0	heterozygous	VUS
APOB	NM_000384.2	2	21236337	rs142798172	C>C/A	p.G1304V	missense	0.00001989	heterozygous	VUS
ALMS1	NM_015120.4	2	73675872	rs752375658	C>C/T	p.L739F	missense	0.000004016	heterozygous	VUS
DES	NM_001927.3	2	220283548	novel	T>C/C	p.Y122H	missense	0	homozygous	Pathogenic
HFE	NM_000410.3	6	26093125	rs140080192	G>G/A	p.E277K	missense	0.003232	heterozygous	VUS
LAMA4	NM_001105206.2	6	112460365	rs41289902	C>C/T	p.R1080Q	missense	0.01031	heterozygous	VUS
KCNQ1	NM_000218.2	11	2683266	novel	A>A/G	p.D490G	missense	0	heterozygous	VUS
FBN1	NM_000138.4	15	48764778	rs377338217	C>C/T	p.V1436M	missense	0.0005164	heterozygous	VUS
LMF1	NM_022773.2	16	919894	rs181731943	C>C/T	p.A469T	missense	0.0007967	heterozygous	VUS

[1] Human genome assembly GRCh37; [2] https://gnomad.broadinstitute.org, October 2019.

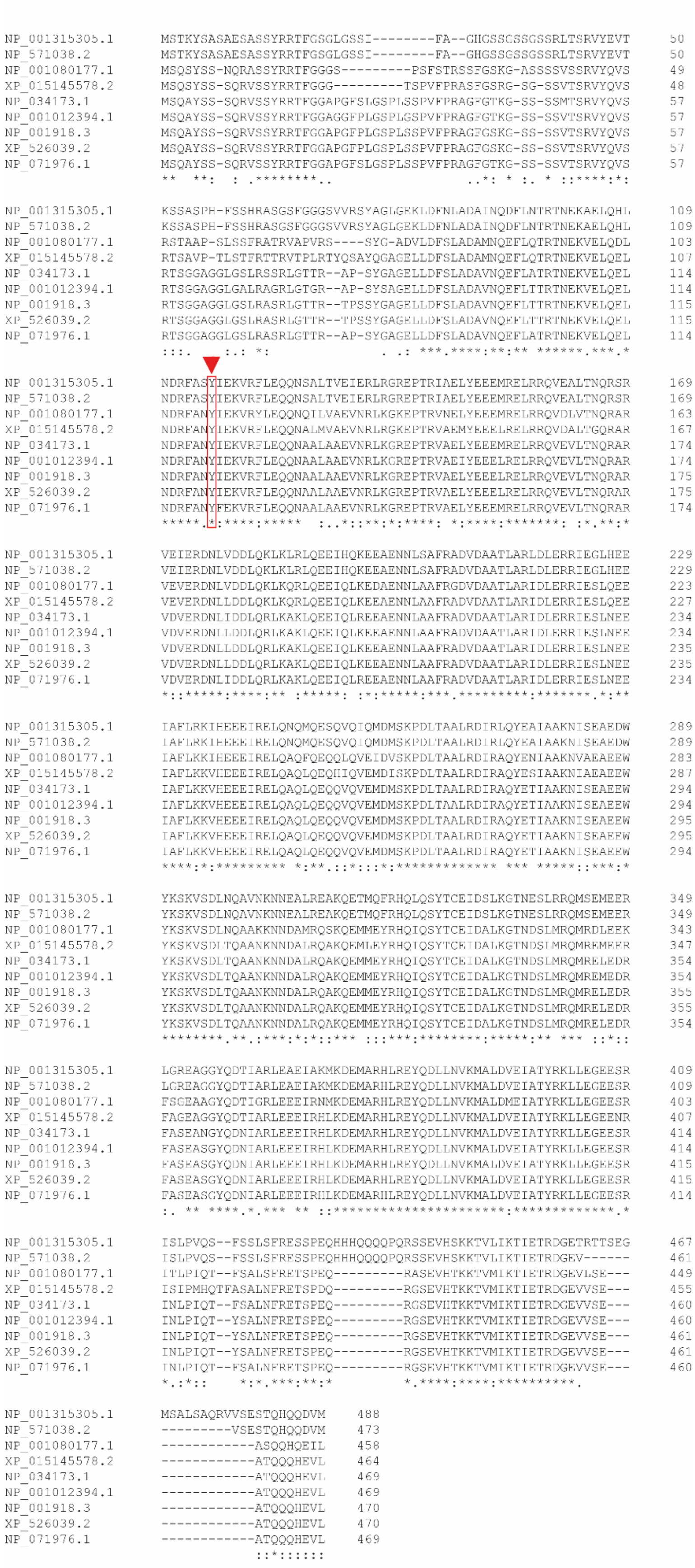

Figure S1. Multiple protein alignments of desmin among different vertebrates. Of note, Y122 is highly conserved between species (red box and arrow).
